# Maternal Fructose Diet-Induced Developmental Programming

**DOI:** 10.3390/nu13093278

**Published:** 2021-09-20

**Authors:** Michael D. Thompson, Brian J. DeBosch

**Affiliations:** 1 Department of Pediatrics, Division of Endocrinology and Diabetes, Washington University School of Medicine, St. Louis, MO 63110, USA; 2 Department of Pediatrics, Division of Gastroenterology, Washington University School of Medicine, St. Louis, MO 63110, USA; deboschb@wustl.edu

**Keywords:** fructose, metabolism, obesity, hypertension, metabolic syndrome, diabetes, uric acid, developmental origins hypothesis of adult disease (DOHaD)

## Abstract

Developmental programming of chronic diseases by perinatal exposures/events is the basic tenet of the developmental origins hypothesis of adult disease (DOHaD). With consumption of fructose becoming more common in the diet, the effect of fructose exposure during pregnancy and lactation is of increasing relevance. Human studies have identified a clear effect of fructose consumption on maternal health, but little is known of the direct or indirect effects on offspring. Animal models have been utilized to evaluate this concept and an association between maternal fructose and offspring chronic disease, including hypertension and metabolic syndrome. This review will address the mechanisms of developmental programming by maternal fructose and potential options for intervention.

## 1. Introduction

The developmental origins hypothesis of adult disease (DOHaD) supports that in utero and perinatal events prime risk for the development of chronic disease later in life. While initial DOHaD studies focused on the effect of undernutrition and subsequent low birth weight, there is now an interest in maternal overnutrition given the current prevalence of pre-pregnancy obesity. The impact of maternal obesity and maternal high fat diet exposure has been extensively studied through birth cohorts and animal models. It is clear that maternal high fat diet exposure programs risk the development of a variety of chronic diseases in offspring, including obesity, cardiovascular disease, cancer, and non-alcoholic fatty liver disease. A common component of an obesogenic diet is fructose; however, much less is known about the impact of maternal fructose exposure and the resultant effects on developmental programming in the offspring. This review will focus specifically on the impact of maternal fructose consumption, as developmental programming by maternal overnutrition has been reviewed elsewhere [1,2].

## 2. Perinatal Fructose Intake and Impact on Pregnancy

A systematic meta-analysis of human studies revealed that fructose consumption was positively associated with increased fasting blood sugar, elevated triglycerides, and elevated systolic blood pressure [3,4]. However, the impact of excessive fructose intake during pregnancy on both fetal and maternal health in humans is largely unknown. In a large prospective cohort study evaluating artificially sweetened and sugar sweetened beverage consumption during pregnancy, an increased risk for preterm delivery was found [5]. It is hypothesized that fructose consumption has deleterious effects on the developing offspring, given the known effects of direct consumption. However, to date, no long-term study has evaluated the effect of perinatal fructose consumption during pregnancy on offspring disease risk.

**Figure 1:**
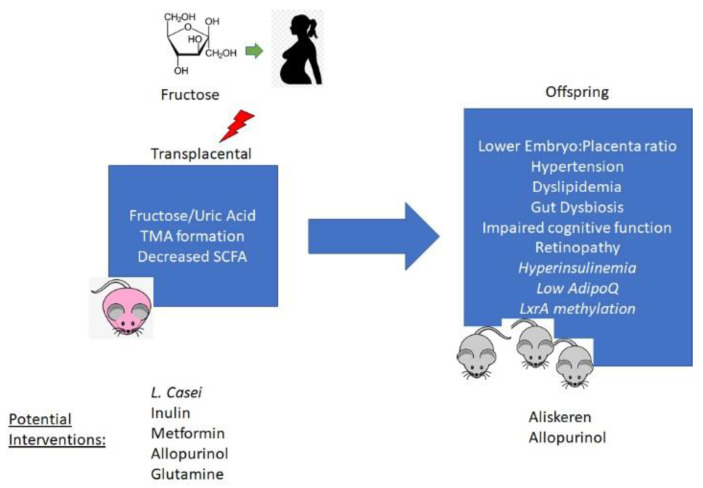
Mechanisms of developmental programming of offspring phenotypes by maternal fructose exposure and potential options for preventive interventions. TMA—trimethylamine; SCFA—short chain fatty acids; LxrA—Liver-X-receptor alpha.

Given this limitation, much of what is known about maternal fructose consumption is with experimental animal models (Figure 1). In rats, consumption of high fructose (HF) during pregnancy resulted in glucose intolerance and hepatic steatosis in the mother [6]. Maternal fructose consumption also decreased placental vascularization, which may result in impaired perfusion of the developing fetus [7]. There may also be a direct effect of fructose on the fetus, as fructose is transported across the placenta [8]. GLUT9 is the likely mediator of this placental transport [9], in light of prior evidence demonstrating its role in urate transport and metabolism in the enterocyte [10]. Maternal fructose consumption also decreases pregnancy rates and litter sizes in mice [11]. This finding was associated with decreased uterine size and impaired decidualization [11].

It is possible that both effects on the pregnant mother as well as direct transplacental passage of fructose confer indirect and direct metabolic risk to the offspring, respectively. In mice, there is a decrease in fetal weight and fetus/placenta ratio that is associated with increased placental uric acid levels [12]. Maternal treatment with allopurinol reversed the changes in fetal growth that were observed, suggesting that fructose metabolism to uric acid is an important mediator of the direct effects of maternal fructose consumption on offspring. Furthermore, serum levels of fructose significantly correlate with uric acid levels in the villous tree of human placenta [12]. Similar to allopurinol, maternal glutamine supplementation in rats decreased placental uric acid levels induced by fructose consumption and abrogated the decrease in fetal weight and length [13]. Further understanding of how fructose-induced increases in placental uric acid production developmentally program phenotypes in offspring will be essential to define the potential for urate production as a target pathway for preventive therapy.

## 3. Maternal Fructose Diet and Offspring Hypertension

A primary focus of research related to maternal fructose consumption and developmental programming in the offspring is on hypertension. Several studies in rodent models have identified an association between maternal HF exposure and increased systolic blood pressure in both male and female offspring [14,15,16]. Notably, the offspring develop hypertension despite consuming a standard chow diet. A primary mechanism for increased hypertension in the offspring involves dysregulated activation of the renin angiotensin aldosterone system (RAAS) [14,15]. This includes increased serum levels of renin, angiotensin, and aldosterone. Maternal HF diet also significantly affects the renal transcriptome with 356 differentially expressed genes identified, several of which are associated with hypertension [17].

Seong, et al. demonstrated that developmental programming of hypertension occurs in an intergenerational manner as both F1 and F2 generation offspring developed elevated systolic blood pressure. The effect is not transgenerational, however, as systolic BP normalized in both F3 and F4 generation offspring. Interestingly, F3 generation offspring still had elevated levels of serum renin, so it may be possible that exposure to a hypertension-inducing stimuli may result in a more severe increase in systolic BP in third generation offspring. A potential mechanism for these changes involves epigenetic modifications in the prorenin receptor promoter [18]. Specifically, there are increases in active histone codes, such as H3Ac and H3K4me2, in the PRR promoter. Conversely, there are decreases in repressive histone codes, including H3K9me3 and H3K27me3. These changes are present across three generations of offspring.

In addition to programming hypertension in adult offspring at baseline, exposure to maternal HF diet worsens high-salt intake-induced hypertension in offspring. Male rat offspring of dams fed a 60% HF diet during pregnancy and lactation developed more severe hypertension after high salt diet feeding compared to offspring from control dams [15]. Increased systolic blood pressure levels were associated with altered expression of factors involved in the renin-angiotensin system, as well as sodium transporters in the kidney.

Several approaches have been evaluated to target the developmental programming of hypertension by maternal fructose. Trimethylamine is a gut microbial metabolite that is linked to hypertension [19,20]. 3,3-dimethyl—butanol inhibits TMA formation and reduces maternal fructose-driven hypertension in offspring [21]. Short chain fatty acids may also be involved as treatment with acetate decreased plasma TMA and protected offspring from maternal fructose-induced hypertension. Another approach to treatment may include targeting of the microbiome with pre- and/or pro-biotics. Maternal treatment with *Lactobacillus casei* or inulin resulted in a significant improvement in maternal HF-induced hypertension [22]. Both treatments modulated serum levels of SCFAs. *Lactobacillus casei* decreased acetate, whereas inulin increased propionate. Both treatments also significantly increased the abundance of *Akkermansia muciniphila.* The abundance of *Akkermansia* has been linked to improvements in blood pressure, and direct *Akkermansia* administration improved several metabolic parameters in a randomized, double-blind, placebo-controlled pilot study in overweight/obese, insulin-resistant volunteers [23,24].

Given the noted alterations in the offspring RAAS after maternal HF diet exposure, compounds that can modify this system could be beneficial. Treatment of offspring with the renin antagonist aliskiren between 2 and 4 weeks of age attenuated the increase in blood pressure induced by maternal HF [25]. Aliskiren increased protein levels of ACE2 and MAS1 receptor in female offspring, suggesting a sex-specific effect on the ACE2/Angiotensin-(1-7)/MAS Axis of the Renin-Angiotensin System. Treatment with prenatal metformin also downregulates the RAAS and prevents maternal fructose-induced hypertension in the offspring [26]. Prenatal metformin decreased markers of renal oxidative stress and serum levels of uric acid in offspring. Maternal metformin may also improve glucose tolerance in offspring [27].

## 4. Maternal Fructose and Offspring Glucose and Lipid Metabolism

Direct exposure to fructose has a well-described effect on hepatic metabolism and the development of hepatic steatosis (reviewed in [28]). There is also an impact of maternal fructose consumption on hepatic lipid metabolism in the offspring. Ten-day-old rat offspring of dams exposed to HF diet exhibit increased hepatic triglyceride levels and changes in the expression of genes involved in beta-oxidation [29]. Seven-month-old adult offspring also exhibit increased steatosis, intrahepatic triglyceride content, and serum transaminases [30]. Altered expression of several clock genes is present in liver of maternal HF offspring [29]. Markers of hepatic inflammation also increase in offspring following maternal HF diet exposure [31]. Similarly, HF feeding in guinea pigs prior to—and during—pregnancy resulted in offspring with significantly altered serum free fatty acids, and increased levels of uric acid and triglycerides, all of which developed before weaning [32].

While it is clear that maternal HF diet affects hepatic lipid metabolism in rodent offspring, the mechanism for this developmental programming has yet to be defined. One potential link may be via altered uric acid metabolism due to maternal fructose consumption. As noted above, maternal fructose increases placental uric acid and fructose transport via GLUT9. GLUT9 knockout in enterocytes induces hyperuricemia, which is associated with the development of increased hepatic triglycerides in mice [10]. This increase in hepatic TG was attenuated by treatment with allopurinol. If maternal fructose exposure increases offspring uric acid levels, this could be a pathway to developing steatosis in offspring. Further understanding of how maternal fructose consumption affects offspring uric acid metabolism is required to directly define any links between developmentally programmed uric acid metabolism and hepatic steatosis in offspring.

Maternal fructose programming of offspring metabolism may occur in a sex-specific manner, with male sex as an apparent predisposing factor to maternally derived metabolic disease. For example, fructose exposure during pregnancy leads to hyperinsulinemia, impaired insulin signaling, and low adiponectin levels in male—but not female—offspring [33]. Furthermore, insulin resistance occurs in male offspring only after maternal fructose consumption [34]. Cholesterol metabolism is also affected in a sex-dependent manner as well, which is likely due to epigenetic regulation of *Lxra* [35]. Promoter methylation at this gene is increased in male offspring, whereas it is decreased in female offspring after maternal fructose exposure. *Lxra* expression may also be affected by maternal fructose-induced changes in miR-206 expression in offspring liver [36].

## 5. Maternal Fructose Effect on Offspring Cognition and Retinopathy

Another area of interest is the effect of maternal fructose consumption of cognitive function in the offspring. Female offspring exposed to maternal fructose had impaired spatial learning and memory [37]. This was associated with decreased hippocampal brain-derived neurotrophic factor (BDNF), and an increase in histone deacetylase 4 (HDAC4) in the nucleus of hippocampal neurons. Stimulation through enriched housing resolved the noted impairments and attenuated the differences observed in hippocampal BDNF levels and HDAC4 distribution [37]. Hippocampal inflammation is also induced in female offspring exposed to maternal fructose via the PPARγ-induced suppression of NFkB signaling [38]. PPARγ also has a role in regulating the maternal fructose-induced suppression of hippocampal astrocyte glycolytic capacity and mitochondrial respiration [39]. These findings suggest that PPARγ may be a target for maternal fructose-induced cognitive deficits. Maternal fructose also alters brain mitochondrial function, notably reducing phosphorylation efficiency [40]. Epigenetic modifications in the hippocampus have been reported following maternal fructose exposure. DNA methylation in the *Cat* promoter is increased in the hippocampus of offspring exposed to maternal HF, with an associated decrease in catalase protein levels [41]. Similarly, maternal HF increased DNA methylation in the promoter of two oxidative stress-associated genes, *Ucp5* and *Tfam* [42]. Further studies will be important to identify additional epigenetic modifications in the brain after maternal HF.

In addition to an effect on neurodevelopment, maternal HF diet may program early-onset retinopathy in offspring. HF consumption during pregnancy and lactation in rats led to an altered electroretinogram in three-month-old female offspring [43]. In particular, the b wave amplitude was decreased with an associated decrease in retinal synaptic plasticity. Interestingly, both changes were prevented by maternal consumption of coenzyme Q10 via an improvement in mitochondrial biogenesis and bioenergetics [43].

## 6. Maternal Fructose Diet and Offspring Microbiome

Another potential mechanism for the transmission of phenotypes driven by maternal diet is though vertical transmission of an altered microbiome. The initial colonizers of the offspring gut are related to the maternal microbiome as the offspring are exposed during delivery and during the lactation period. The metabolic significance of a “healthy” microbiome in the perinatal period has been well described (reviewed in [44]). HF diet feeding significantly alters the composition and metabolic profile of the gut microbiome in mice [45]. These changes are detectable as early as 7 days of HF diet feeding. It is also evident that the maternal microbiome changes after fructose exposure. The addition of 10% fructose to the drinking water of female rats before and during pregnancy significantly altered the maternal microbiome [46]. Notably, there was a significant reduction in *Lactobacillus* and *Bacteroides*. This finding was associated with a reduction in the expression of genes linked to gut barrier integrity in the offspring; however, the offspring microbiome was not assessed.

Similarly, maternal HF diet also alters the microbiome in rat offspring. Hsu et. al. showed that maternal HF diet exposure was associated with significantly decreased abundance of genus *Alkaliphilus* and increased abundance of genus *Lactobacillus* [22]. At the species level, *Bacteroides acidifaciens* was increased in maternal HF-exposed offspring. Notably, these changes were normalized by treatment with prebiotics (inulin) or probiotics (*Lactobacillus casei)*. A follow-up study identified that maternal fructose is associated with decreased alpha-diversity in the offspring microbiome, a decrease in the abundance of *Lactobacillus,* and increased abundance of *Akkermansia* [47].

## 7. Areas for Future Study

With an increase in the consumption of fructose in the diet, understanding the impact of such exposure in the perinatal period has clearly become important. Current studies show a link between maternal fructose exposure and the development of hypertension, metabolic liver disease, and altered cognition in the offspring. While some studies have provided evidence for this mechanism via epigenetic modifications and alterations of the offspring microbiome, there is an opportunity and need for further analysis of the mechanism to define how maternal fructose consumption drives phenotypes in the offspring. There are several other areas for future study as the field moves forward. Very little is known about the developmental programming impact of paternal fructose consumption. Fructose consumption does have a clear impact on sperm production and testicular development during puberty [48]. This exposure may also alter the epigenetic architecture of sperm DNA, which could have consequences for future offspring. Whereas most studies have explored the effect of fructose exposure during pregnancy, it is unclear if there is an impact of preconception fructose consumption. It will be important to identify if fructose consumption has an effect on oocyte development and whether there is an induction of epigenetic changes in the oocyte. At this point, most studies evaluating the effect of maternal fructose exposure have only examined the first generation. Understanding the inter- and trans-generational effect of this perinatal exposure will likely provide evidence for lasting epigenetic changes. Lastly, while some studies have begun to evaluate potential interventions for phenotypes induced by perinatal fructose exposure, deeper mechanistic studies, using the described experimental models, will identify novel targets to evaluate.

## References

[1] Hanson M.A., Gluckman P.D. (2014). Early developmental conditioning of later health and disease: Physiology or pathophysiology?. Physiol. Rev..

[2] Fleming T.P., Watkins A.J., Velazquez M.A., Mathers J.C., Prentice A.M., Stephenson J., Barker M., Saffery R., Yajnik C.S., Eckert J.J. (2018). Origins of lifetime health around the time of conception: Causes and consequences. Lancet.

[3] Jia G., Hill M.A., Sowers J.R. (2019). Maternal Exposure to High Fructose and Offspring Health. Hypertension.

[4] Kelishadi R., Mansourian M., Heidari-Beni M. (2014). Association of fructose consumption and components of metabolic syndrome in human studies: A systematic review and meta-analysis. Nutrition.

[5] Englund-Ogge L., Brantsaeter A.L., Haugen M., Sengpiel V., Khatibi A., Myhre R., Myking S., Meltzer H.M., Kacerovsky M., Nilsen R.M. (2012). Association between intake of artificially sweetened and sugar-sweetened beverages and preterm delivery: A large prospective cohort study. Am. J. Clin. Nutr..

[6] Zou M., Arentson E.J., Teegarden D., Koser S.L., Onyskow L., Donkin S.S. (2012). Fructose consumption during pregnancy and lactation induces fatty liver and glucose intolerance in rats. Nutr. Res..

[7] Alzamendi A., Del Zotto H., Castrogiovanni D., Romero J., Giovambattista A., Spinedi E. (2012). Oral metformin treatment prevents enhanced insulin demand and placental dysfunction in the pregnant rat fed a fructose-rich diet. ISRN Endocrinol..

[8] Hagerman D.D., Villee C.A. (1952). The transport of fructose by human placenta. J. Clin. Investig..

[9] Bibee K.P., Illsley N.P., Moley K.H. (2011). Asymmetric syncytial expression of GLUT9 splice variants in human term placenta and alterations in diabetic pregnancies. Reprod. Sci..

[10] DeBosch B.J., Kluth O., Fujiwara H., Schurmann A., Moley K. (2014). Early-onset metabolic syndrome in mice lacking the intestinal uric acid transporter SLC2A9. Nat. Commun..

[11] Saben J.L., Asghar Z., Rhee J.S., Drury A., Scheaffer S., Moley K.H. (2016). Excess Maternal Fructose Consumption Increases Fetal Loss and Impairs Endometrial Decidualization in Mice. Endocrinology.

[12] Asghar Z.A., Thompson A., Chi M., Cusumano A., Scheaffer S., Al-Hammadi N., Saben J.L., Moley K.H. (2016). Maternal fructose drives placental uric acid production leading to adverse fetal outcomes. Sci. Rep..

[13] Olaniyi K.S., Sabinari I.W., Olatunji L.A. (2020). Oral L-glutamine rescues fructose-induced poor fetal outcome by preventing placental triglyceride and uric acid accumulation in Wistar rats. Heliyon.

[14] Seong H.Y., Cho H.M., Kim M., Kim I. (2019). Maternal High-Fructose Intake Induces Multigenerational Activation of the Renin-Angiotensin-Aldosterone System. Hypertension.

[15] Tain Y.L., Lee W.C., Leu S., Wu K., Chan J. (2015). High salt exacerbates programmed hypertension in maternal fructose-fed male offspring. Nutr. Metab. Cardiovasc. Dis..

[16] Tain Y.L., Leu S., Wu K.L., Lee W.C., Chan J.Y. (2014). Melatonin prevents maternal fructose intake-induced programmed hypertension in the offspring: Roles of nitric oxide and arachidonic acid metabolites. J. Pineal Res..

[17] Tain Y.L., Hsu C.N., Chan J.Y., Huang L.T. (2015). Renal Transcriptome Analysis of Programmed Hypertension Induced by Maternal Nutritional Insults. Int. J. Mol. Sci..

[18] Cho H.M., Kim I. (2020). Maternal high-fructose intake induces hypertension through activating histone codes on the (pro)renin receptor promoter. Biochem. Biophys. Res. Commun..

[19] Brunt V.E., Casso A.G., Gioscia-Ryan R.A., Sapinsley Z.J., Ziemba B.P., Clayton Z.S., Bazzoni A.E., VanDongen N.S., Richey J.J., Hutton D.A. (2021). Gut Microbiome-Derived Metabolite Trimethylamine N-Oxide Induces Aortic Stiffening and Increases Systolic Blood Pressure With Aging in Mice and Humans. Hypertension.

[20] Brunt V.E., Gioscia-Ryan R.A., Casso A.G., VanDongen N.S., Ziemba B.P., Sapinsley Z.J., Richey J.J., Zigler M.C., Neilson A.P., Davy K.P. (2020). Trimethylamine-N-Oxide Promotes Age-Related Vascular Oxidative Stress and Endothelial Dysfunction in Mice and Healthy Humans. Hypertension.

[21] Hsu C.N., Chang-Chien G.P., Lin S., Hou C.Y., Tain Y.L. (2019). Targeting on Gut Microbial Metabolite Trimethylamine-N-Oxide and Short-Chain Fatty Acid to Prevent Maternal High-Fructose-Diet-Induced Developmental Programming of Hypertension in Adult Male Offspring. Mol. Nutr. Food Res..

[22] Hsu C.N., Lin Y.J., Hou C.Y., Tain Y.L. (2018). Maternal Administration of Probiotic or Prebiotic Prevents Male Adult Rat Offspring against Developmental Programming of Hypertension Induced by High Fructose Consumption in Pregnancy and Lactation. Nutrients.

[23] Roshanravan N., Mahdavi R., Alizadeh E., Ghavami A., Rahbar Saadat Y., Mesri Alamdari N., Alipour S., Dastouri M.R., Ostadrahimi A. (2017). The effects of sodium butyrate and inulin supplementation on angiotensin signaling pathway via promotion of Akkermansia muciniphila abundance in type 2 diabetes; A randomized, double-blind, placebo-controlled trial. J. Cardiovasc. Thorac. Res..

[24] Depommier C., Everard A., Druart C., Plovier H., Van Hul M., Vieira-Silva S., Falony G., Raes J., Maiter D., Delzenne N.M. (2019). Supplementation with Akkermansia muciniphila in overweight and obese human volunteers: A proof-of-concept exploratory study. Nat. Med..

[25] Hsu C.N., Wu K.L., Lee W.C., Leu S., Chan J.Y., Tain Y.L. (2016). Aliskiren Administration during Early Postnatal Life Sex-Specifically Alleviates Hypertension Programmed by Maternal High Fructose Consumption. Front. Physiol..

[26] Tain Y.L., Wu K.L.H., Lee W.C., Leu S., Chan J.Y.H. (2018). Prenatal Metformin Therapy Attenuates Hypertension of Developmental Origin in Male Adult Offspring Exposed to Maternal High-Fructose and Post-Weaning High-Fat Diets. Int. J. Mol. Sci..

[27] Gregg B.E., Botezatu N., Brill J.D., Hafner H., Vadrevu S., Satin L.S., Alejandro E.U., Bernal-Mizrachi E. (2018). Gestational exposure to metformin programs improved glucose tolerance and insulin secretion in adult male mouse offspring. Sci. Rep..

[28] Federico A., Rosato V., Masarone M., Torre P., Dallio M., Romeo M., Persico M. (2021). The Role of Fructose in Non-Alcoholic Steatohepatitis: Old Relationship and New Insights. Nutrients.

[29] Clayton Z.E., Vickers M.H., Bernal A., Yap C., Sloboda D.M. (2015). Early Life Exposure to Fructose Alters Maternal, Fetal and Neonatal Hepatic Gene Expression and Leads to Sex-Dependent Changes in Lipid Metabolism in Rat Offspring. PLoS ONE.

[30] Koo S., Kim M., Cho H.M., Kim I. (2021). Maternal high-fructose intake during pregnancy and lactation induces metabolic syndrome in adult offspring. Nutr. Res. Pract..

[31] Carapeto P.V., Ornellas F., Mandarim-de-Lacerda C.A., Aguila M.B. (2018). Liver metabolism in adult male mice offspring: Consequences of a maternal, paternal or both maternal and paternal high-fructose diet. J. Dev. Orig. Health Dis..

[32] Smith E.V.L., Dyson R.M., Berry M.J., Gray C. (2020). Fructose Consumption During Pregnancy Influences Milk Lipid Composition and Offspring Lipid Profiles in Guinea Pigs. Front. Endocrinol..

[33] Rodriguez L., Panadero M.I., Roglans N., Otero P., Rodrigo S., Alvarez-Millan J.J., Laguna J.C., Bocos C. (2016). Fructose only in pregnancy provokes hyperinsulinemia, hypoadiponectinemia, and impaired insulin signaling in adult male, but not female, progeny. Eur. J. Nutr..

[34] Rodriguez L., Otero P., Panadero M.I., Rodrigo S., Alvarez-Millan J.J., Bocos C. (2015). Maternal fructose intake induces insulin resistance and oxidative stress in male, but not female, offspring. J. Nutr. Metab..

[35] Rodrigo S., Fauste E., de la Cuesta M., Rodriguez L., Alvarez-Millan J.J., Panadero M.I., Otero P., Bocos C. (2018). Maternal fructose induces gender-dependent changes in both LXRalpha promoter methylation and cholesterol metabolism in progeny. J. Nutr. Biochem..

[36] Yamazaki M., Munetsuna E., Yamada H., Ando Y., Mizuno G., Fujii R., Nouchi Y., Kageyama I., Teshigawara A., Ishikawa H. (2020). Maternal fructose consumption down-regulates Lxra expression via miR-206-mediated regulation. J. Nutr. Biochem..

[37] Wu K.L., Wu C.W., Tain Y.L., Huang L.T., Chao Y.M., Hung C.Y., Wu J.C., Chen S.R., Tsai P.C., Chan J.Y. (2016). Environmental stimulation rescues maternal high fructose intake-impaired learning and memory in female offspring: Its correlation with redistribution of histone deacetylase 4. Neurobiol. Learn. Mem..

[38] Liu W.C., Wu C.W., Fu M.H., Tain Y.L., Liang C.K., Hung C.Y., Chen I.C., Lee Y.C., Wu C.Y., Wu K.L.H. (2020). Maternal high fructose-induced hippocampal neuroinflammation in the adult female offspring via PPARgamma-NF-kappaB signaling. J. Nutr. Biochem..

[39] Wu C.W., Hung C.Y., Hirase H., Tain Y.L., Lee W.C., Chan J.Y.H., Fu M.H., Chen L.W., Liu W.C., Liang C.K. (2019). Pioglitazone reversed the fructose-programmed astrocytic glycolysis and oxidative phosphorylation of female rat offspring. Am. J. Physiol. Endocrinol. Metab..

[40] Mortensen O.H., Larsen L.H., Orstrup L.K., Hansen L.H., Grunnet N., Quistorff B. (2014). Developmental programming by high fructose decreases phosphorylation efficiency in aging offspring brain mitochondria, correlating with enhanced UCP5 expression. J. Cereb. Blood Flow Metab..

[41] Mizuno G., Munetsuna E., Yamada H., Yamazaki M., Ando Y., Hattori Y., Kageyama I., Teshigawara A., Nouchi Y., Fujii R. (2021). Maternal fructose consumption downregulates hippocampal catalase expression via DNA methylation in rat offspring. Nutr. Res..

[42] Yamada H., Munetsuna E., Yamazaki M., Mizuno G., Sadamoto N., Ando Y., Fujii R., Shiogama K., Ishikawa H., Suzuki K. (2019). Maternal fructose-induced oxidative stress occurs via Tfam and Ucp5 epigenetic regulation in offspring hippocampi. FASEB J..

[43] Huang H.M., Wu C.W., Chen I.C., Lee Y.C., Huang Y.S., Hung C.Y., Wu K.L.H. (2021). Maternal high-fructose diet induced early-onset retinopathy via the suppression of synaptic plasticity mediated by mitochondrial dysfunction. Am. J. Physiol. Endocrinol. Metab..

[44] Mirpuri J., Neu J. (2021). Maternal microbial factors that affect the fetus and subsequent offspring. Semin. Perinatol..

[45] Montrose D.C., Nishiguchi R., Basu S., Staab H.A., Zhou X.K., Wang H., Meng L., Johncilla M., Cubillos-Ruiz J.R., Morales D.K. (2021). Dietary Fructose Alters the Composition, Localization, and Metabolism of Gut Microbiota in Association With Worsening Colitis. Cell. Mol. Gastroenterol. Hepatol..

[46] Astbury S., Song A., Zhou M., Nielsen B., Hoedl A., Willing B.P., Symonds M.E., Bell R.C. (2018). High Fructose Intake During Pregnancy in Rats Influences the Maternal Microbiome and Gut Development in the Offspring. Front. Genet..

[47] Hsu C.N., Chan J.Y.H., Wu K.L.H., Yu H.R., Lee W.C., Hou C.Y., Tain Y.L. (2021). Altered Gut Microbiota and Its Metabolites in Hypertension of Developmental Origins: Exploring Differences between Fructose and Antibiotics Exposure. Int. J. Mol. Sci..

[48] Medaglia D.S.A., Vieira H.R., Silveira S.D.S., Siervo G., Marcon M., Mathias P.C.F., Fernandes G.S.A. (2021). High-fructose diet during puberty alters the sperm parameters, testosterone concentration, and histopathology of testes and epididymis in adult Wistar rats. J. Dev. Orig. Health Dis..

